# Effects of pre-pregnancy body mass index on cardiometabolic biomarkers in pregnant emirati women

**DOI:** 10.1186/s12978-024-01940-9

**Published:** 2024-12-18

**Authors:** Lolowa A. Almekhaini, Shamsa A. Awar, Taoufik Zoubeidi, Sania Al Hamad, Javed Yasin, Junu V. George, Maha Khaled, Nehaya Qasem, Fatima Bahwan, Hassib Narchi, Elhadi H. Aburawi

**Affiliations:** 1 Departments of Pediatrics, United Arab Emirates University, Al-Ain, United Arab Emirates; 2 Obstetrics and Gynecology, United Arab Emirates University, Al-Ain, United Arab Emirates; 3 Biostatistics, United Arab Emirates University, Al-Ain, United Arab Emirates; 4 Internal Medicine, United Arab Emirates University, Al-Ain, United Arab Emirates; 5 Mediclinic Middle East, Dubai, United Arab Emirates; 6 Tawam Hospitals, Al-Ain- Abu Dhabi, United Arab Emirates; 7 Undergraduate student, College of Medicine and Health Sciences, United Arab Emirates University, Al-Ain, United Arab Emirates

**Keywords:** Pregnancy, Obesity, Inflammation, Cardiometabolic biomarkers

## Abstract

**Background:**

To study effect of pregnancy on obese women’s maternal cardiometabolic biomarkers as a signature for maternal morbidity and complications.

**Methods:**

This cross-sectional cohort study involved pregnant Emirati women who had regular menstrual cycles and had normal blood pressure. Pre-pregnancy body mass index was calculated using height and weight measurements recorded within three months before current pregnancy. Average systolic and diastolic blood pressure measurements were calculated from each visit. Blood samples were collected randomly once and following cardiometabolic biomarkers were measured.

**Results:**

We enrolled 178 pregnant women, with a mean age ± standard deviation of cohort was 29.9 ± 4.97 years and Pre-pregnancy body mass index 28.11 ± 6.58 kg/m2. None of blood pressure measurements or biomarkers serum concentrations were statistically different across Pre-pregnancy body mass index groups except for soluble intercellular cytoadhesive molecule-1levels which were the highest in underweight women. Pregnant women with pre-gestational obesity had higher systolic and diastolic blood pressure levels compared to women with normal or overweight. All variables were statistically significantly different by trimesters except systolic blood pressure, random blood glucose, lipoprotein-A, and high-sensitivity C-Reactive Protein. After adjusting, in a multivariate linear regression model, for maternal age, trimester of pregnancy, education level, parity and smoking, none of biomarkers or blood pressure were found to be significantly associated with Pre-pregnancy body mass index. In a multivariate linear regression model adjusting for maternal age, Pre-pregnancy body mass index, education level, parity and smoking, gamma-glutamyl transferase, total cholesterol, high density lipoprotein, low-density lipoprotein, triglycerides, apolipoprotein A & B, interleukin-6, tumor necrosis factor-alpha and insulin-like growth factor-1 concentrations remained significantly associated with advancing trimester of pregnancy. There was a significant interaction between Pre-pregnancy body mass index and trimester of pregnancy for serum gamma-glutamyl transferase and soluble intercellular cytoadhesive molecule-1concentration.

**Conclusion:**

This study emphasizes how pregnancy has a significant impact on cardiometabolic markers in obese women, indicating both hyperlipidemic status of pregnancy and diabetogenic tendency in obese patients who are not diabetics. These findings may suggest that pregnancy in obese patients increases risk of developing metabolic syndrome in future, therefore more attention is recommended of pregnant obese women and further study of establishing specific cardiometabolic biomarkers screening program.

## Plain Language Summary

The World Health Organization (WHO) has recommended a prevention strategy to reduce the maternal complications of obese pregnant women. This study assesses the effect of pregnancy on obese women’s maternal cardiometabolic biomarkers (CMBs) as risk of increase maternal morbidity and complications. The women Pre-pregnancy body mass index (pBMI) was calculated and their blood pressure measured. Blood samples were collected randomly once during maternal clinical visits and the cardiometabolic biomarkers were analyzed. Many variables were significantly different by pregnancy trimesters that emphasizes the negative impact of pregnancy on cardiometabolic markers in obese women. These findings may suggest that pregnancy in obese patients increases the risk of developing metabolic syndrome in the future.

## Introduction

With the increase in body mass index (BMI) in women of reproductive age, the World Health Organization (WHO) has recommended a prevention strategy aimed at reducing associated risks of maternal complications [1].

Maternal pre-pregnancy obesity, defined as a BMI ≥ 30 kg/m^2^, is recognized as one of factors that negatively affect maternal health [2]). Although the precise mechanisms with which maternal obesity led to adverse outcomes are not yet fully elucidated, it is likely that associated changes in metabolic, inflammatory and cardiovascular biomarkers during pregnancy play a major role [3–7]. Maternal obesity has been proven to increase the risk of co-morbidities such as insulin resistance, pregnancy induced hypertension, increased rates of fetal anomalies and abnormal fetal growth, as well as the rates of instrumented vaginal deliveries and cesarean sections [8]. Severe maternal morbidity has also been associated with an immediate and late elevated risk of severe maternal morbidity and/or mortality [9].

Metabolic factors play an essential role. Blood hemoglobin A1c (HbA1c) levels are significantly higher in obese mothers [10, 11]. Complications such as pre-eclampsia, gestational diabetes mellitus, atherosclerosis, preterm delivery, are known to be associated with maternal dyslipidemia and diabetes mellitus [12, 13]. Apoprotein-A (APO-A) and apoprotein-B (APO-B) are involved in lipid transport and their dysregulation is associated with atherosclerosis. APO-A is a protein carried in high-density lipoprotein-C, facilitates its removal, thus helping lower risk for cardiovascular disease. APO-B is a protein involved in lipid transport and is the primary apolipoprotein found in various lipoprotein particles. Elevated levels are associated with an increased risk of developing atherosclerosis, heart and vascular disease. Insulin-like growth factor 1 (IGF-1) plays a crucial role in embryonic and placental development, regulating placenta growth. In pregnancy, the main source of IGF-I is the decidua.

Other mechanisms involved include endothelial dysfunction which is associated with diminished availability of nitric oxide and/or an imbalance in the relative contribution of endothelium-derived relaxing and contracting factors. During pregnancy, endothelial function is critical for placentation, maternal volume expansion, and placental perfusion. Markers of endothelial dysfunction are associated with adverse birth outcomes. Levels of endothelial biomarkers, such as soluble vascular adhesion molecule 1 (sVCAM-1) and intercellular adhesion molecule 1 (ICAM-1) rise following stimulation by interleukins and tumor necrosis factor-α released during inflammation. Soluble intercellular adhesion molecule-1 (sICAM-1), a circulating form of ICAM-1**,** plays a role in neutrophil recruitment and trafficking into various tissues. ICAM-1is expressed on endothelial cells and cells of the immune system. Elevated levels of inflammatory and endothelial dysfunction biomarkers also contribute to chronic inflammation, obesity, atherosclerosis, abnormal fetal growth, preterm delivery, difficulties establishing exclusive breastfeeding after birth, and immune dysregulation in the offspring [1, 3, 6, 7, 14–17]. Inflammation also plays a role in pregnancy outcomes, with elevated serum concentrations of high-sensitivity C-reactive protein (Hs-CRP) in the second trimester of pregnancy demonstrated to be associated with lower birth weight of their offspring [6, 7, 14, 16, 18].

Pre-gestational obese women also tend to have higher arterial blood pressure (BP) compared to those with a normal pre-pregnancy BMI (pBMI), an important additional indicator of maternal cardiovascular complications, including pregnancy-induced hypertension, preeclampsia, preterm birth, and intrauterine growth restriction [3, 12, 13, 19].

The population in the Middle East has higher cardiovascular disease (CVD) risk factors (97%) than rest of world (90.4%), of which, 80% are preventable [20]. Age-adjusted prevalence rate for CVD worldwide in 2020 was 7354.1 per 100,000, compared to 10,148 per 100, 000 in the Middle East. In the United Arab Emirates (UAE), the prevalence of overweight and obesity has been rapidly increasing, particularly among young females of reproductive age [21–25].

the study’s objective and hypothesis are exploring the direct relationship between pBMI and cardiovascular biomarkers in expectant mothers at different stages of pregnancy. Additionally, assessing whether there was any interaction (effect modifier) between the pBMI and the pregnancy trimesters.

## Material and methods

This cross-sectional study enrolled Emirati pregnant mothers who were receiving care at obstetric clinic in two tertiary hospitals located in Al Ain city, namely Al Wagan and Tawam hospitals. They are part of Abu Dhabi Health Services Company, which serves a total population of 600,000. Prior to their participation, each woman was provided with detailed information about study and gave informed written consent.

All participants received regular antenatal care, which included consultations and follow-ups with obstetricians, ultrasound assessments to monitor fetal growth and detect anomalies, education on maternal health and nutrition, guidance on physical activity, and advice on avoiding environmental hazards as per international guidelines, specifically those established by National Institute for Health and Care Excellence (NICE) and American College of Obstetricians and Gynecologists (ACOG).

The study received approval from Tawam Human Research Ethics Committee, with approval number THREC-627.

### Sample size calculation

Previous studies of individual biomarkers during pregnancy have included a relatively small number of participants. This is not suitable for the calculation of the required sample size when several of these biomarkers are simultaneously investigated. However, as only hypertension was consistently reported in several larger studies, we based the calculation of the required sample size on their reported prevalence of gestational hypertension. Based on a recent reported prevalence of gestational hypertension of 13.8% in 137,389 pregnancies [26], the sample size required for this study with an estimated that prevalence ± 5% with a 95% confidence level was calculated as 174 participants (OpenEpi, Version 3, 2024).

### Inclusion criteria

Between June 2019 and May 2022, pregnant Emirati women within age range of 18 to 35 years, with a live singleton pregnancy were enrolled before 12 weeks’ gestation if they had regular menstrual cycles, accurate knowledge of first day of their last menstrual period (LMP), and normal blood pressure. Recruitment process involved random selection, with selection of every second woman visiting either one of two hospitals if they met inclusion criteria.

### Exclusion criteria

Women were excluded from study if they have any known risk factors other than obesity such as current smokers, diabetes mellitus, hypertension, kidney disease, any type of cancer, autoimmune disorders, hematologic diseases, infection with human immunodeficiency virus, hepatic diseases, or pregnancy-associated complications such as gestational diabetes, preeclampsia, multiple gestation or fetal anomaly.

## Maternal characteristics

During the selected antenatal visit for each participant, after obtaining consent, their baseline characteristics were gathered using a standardized questionnaire. They included: maternal age, parity, smoking history, education level, consanguineous marriage, and medical, surgical and obstetric history. Gestational age was confirmed by ultrasound measurement of fetal crown–rump length at 12–13 weeks of gestation. Routine fetal ultrasound measurements were performed in second and third trimesters, or as recommended by obstetrician [21, 27, 28]. Gestational age was categorized into first (0–13 week + 6 days), second (14–27 week + 6 days) and third (28–42 week.) trimester [29]. Mean values of pre-pregnancy weight (kg) and height (cm) of each participant were obtained from their medical record within three months preceding current pregnancy. BMI was calculated [weight (Kg)/height^2^ (m^2^)] and was used to classify participants into four groups, based on US Centers for Disease Control and Prevention (CDC), National Institutes of Health (NIH) and WHO criteria: underweight (< 18.5 kg/m^2^), normal weight (18.5–24.9 kg/m^2^), overweight (25–29.9 kg/m^2^), and obese (≥ 30 kg/m^2^) [30].

## Maternal biochemical markers

A computer-based random selection assigned participants, in each trimester of pregnancy, to submit a single venous sample of whole blood after overnight fasting. Thus, each woman was selected only once throughout her pregnancy. Blood was collected in two vacutainer tubes, one containing EDTA as an anticoagulant, and another plain tube with Gel Clot Activator. Blood samples were thoroughly mixed at room temperature and transferred to laboratory in an ice box. Both tubes were then centrifuged at 3600 RPM for 10 mins and serum and plasma were separated and stored at – 80 ^°^C. Automated analyzer Integra 400 Plus (Roche Diagnostics, Mannheim, Germany) was used for measurements of following blood biomarkers: HbA_1c_, blood glucose, triglycerides (TG), total cholesterol (Total-C), high-density lipoprotein (HDL), low-density lipoprotein (LDL), Apo-A & Apo-B, lipoprotein- a (Lp-a), Hs-CRP and gamma-glutamyl transferase (GGT). Commercially available method enzyme linked immunosorbent assay (ELISA) from R&D Systems from USA was used for measurements of blood levels of (IGF-1, DG100B), interleukin-6 (IL-6, D6050), tumor necrosis factor-alpha (TNF-α, DTA00C), (sICAM-1, DCD540) and (sVCAM-1, DVC00).

### Maternal blood pressure measurements

A manual mercury sphygmomanometer was used to measure BP by trained nurses based on American College of Cardiology and American Heart Association guidelines. For each participant, both systolic and diastolic BP were measured three times during each trimester, resulting in nine measurements of arterial BP during pregnancy. The average systolic BP and diastolic BP measurements in each trimester were used in the analysis.

### Statistical analysis

Data was saved in Microsoft Excel (Office 2019) and analyzed using IBM SPSS Statistics software (version 28.0, IBM Corporation, Armonk, NY, USA) and Stata version 18 (Stata Corps, Texas). Only samples with complete data were included in the analyses and no imputation was performed. The independent or explanatory variables that we studied were pBMI, trimester of pregnancy, age, parity, smoking, and education level. The dependent or outcome variables measured across the three trimesters of pregnancy were hemodynamic (BP) and several biomarkers. The metabolic biomarkers included HbA_1c_, blood glucose, TG, Total-C, HDL, LDL, Apo-A & Apo-B, and Lp-a. Inflammatory markers included Hs-CRP. IL-6, TNF-α and liver biomarker included gamma-glutamyl GGT. Endothelial biomarkers included sICAM-1 and sVCAM-1 serum concentrations. Results of continuous variables were expressed as mean value ± standard deviation (SD) and frequencies as number and percentage.

One-way analysis of variance (ANOVA) was used to compare means of continuous quantitative variables across pBMI groups and trimesters, with post-hoc pairwise comparisons with the Bonferroni test which adjusts the significance level to determine which specific groups differ from each other and reduces the likelihood of Type I errors (false positives). Two-way ANOVA was performed to evaluate the interaction between the independent variables (factors), where the effect on the dependent variable of one factor should be always interpreted in light of the other factor. Variables associated with the outcomes with a p-value <0.10 in the univariate model were included in a linear multivariate regression model to adjust for confounders as well as for interaction. Pearson’s chi-square test was used to compare qualitative variables (e.g., dichotomous variable vitamin D deficiency) across BMI groups. When this test was not valid due to low expected frequencies, Fisher’s Exact test was used instead. For all analyses, a p-value < 0.05 defined statistical significance.

## Results

From one hundred eighty-eight pregnant women initially enrolled in study, ten (5.6 %) were excluded, because of gestational diabetes mellitus and miscarriages. The remaining one hundred seventy-eight women were included in study. The mean age -± standard deviation of the cohort was 29.9 ± 4.97 years. and pBMI 28.11 ± 6.58 kg/m2. The number of participants in each trimester of pregnancy was not statistically significantly different. Except for the pre-pregnancy weight and BMI, there were no significant differences between study participants’ characteristics (age, trimester of pregnancy, consanguinity, and parity) across pBMI groups, except for mother’s education with the highest prevalence of university education in overweight woman (Table 1).

**Table 1:** Characteristics of 178 pregnant women enrolled in analysis by pre-pregnancy body mass index (pBMI)

Variable		Underweight n = 5	Normal weight n = 59	Overweight n = 52	Obese n = 62	Total n = 178	P value
Age (y) mean ± SD		27.4 ± 6.06	28.4 ± 4.23	28.6 ± 4.72	30.0 ± 5.69	29.9 ± 4.97	0.25^a^
Preconception weight (kg) mean ± SD		44.5 ± 7.0	56.5 ± 6.38	67.9 ± 5.00	87.9 ± 13.65	70.3 ± 16.67	< 0.001^a^
Pre-conception height (cm) mean ± SD		163 ± 7.03	158.8 ± 5.45	157.8 ± 4.51	157.5 ± 4.42	158.2 ± 4.93	0.21^a^
Preconception BMI (kg/m2) mean ± SD		16.65 ± 1.17	22.35 ± 1.76	27.22 ± 1.31	33.37 ± 4.98	28.11 ± 6.58	< 0.001
Trimester n (%)	First Second Third	1 (20) 2 (40) 2 (40)	11 (18.6) 23 (39.0) 25 (42.3)	14 (36.9) 16 (30.7) 22 (42.3)	16 (25.8) 28 (45.1) 18 (29.0)	42 69 67	0.58^b^
Consanguinity n (%)	1st Degree 2nd Degree None	0 (0) 0 (0) 0 (0)	21 (35.6) 7 (11.9) 31 (52.5)	14 (6.9) 13 (25.0) 25 (48.0)	21 (33.9) 14 (22.6) 27 (43.5)	56 34 88	0.20^b^
Parity n (%)	Multi Primi	3 (60) 2 (40)	53 (89.8) 6 (10.2)	41 (78.9) 11 (21.1)	52 (83.9) 10 (16.1)	149 29	0.116^**b**^
Mother education n (%)	Less than high school High school University	0 (0) 2 (50) 2 (40)	0 (0) 24 (40.7) 35 (42.5)	0 (0) 28 (38.4) 25 (48.1)	5 (8.0) 29 (37.3) 25 (40.3)	5 75 87	0.02^b^
Smoking n (%)		0 (0)	14 (23.7)	10 (19.2)	12 (19.3)	31	0.65^b^

Underweight (BMI < 18.5 kg/m2), normal weight (BMI 18.5–24.9 kg/m2), overweight (BMI 25–29.9 kg/m2), and obese (BMI ≥ 30 kg/m2); BMI (body mass index)

^a^One-way analysis of variance (ANOVA)

^b^Pearson’s chi-square or Fisher's exact test

**Fig. 1:**
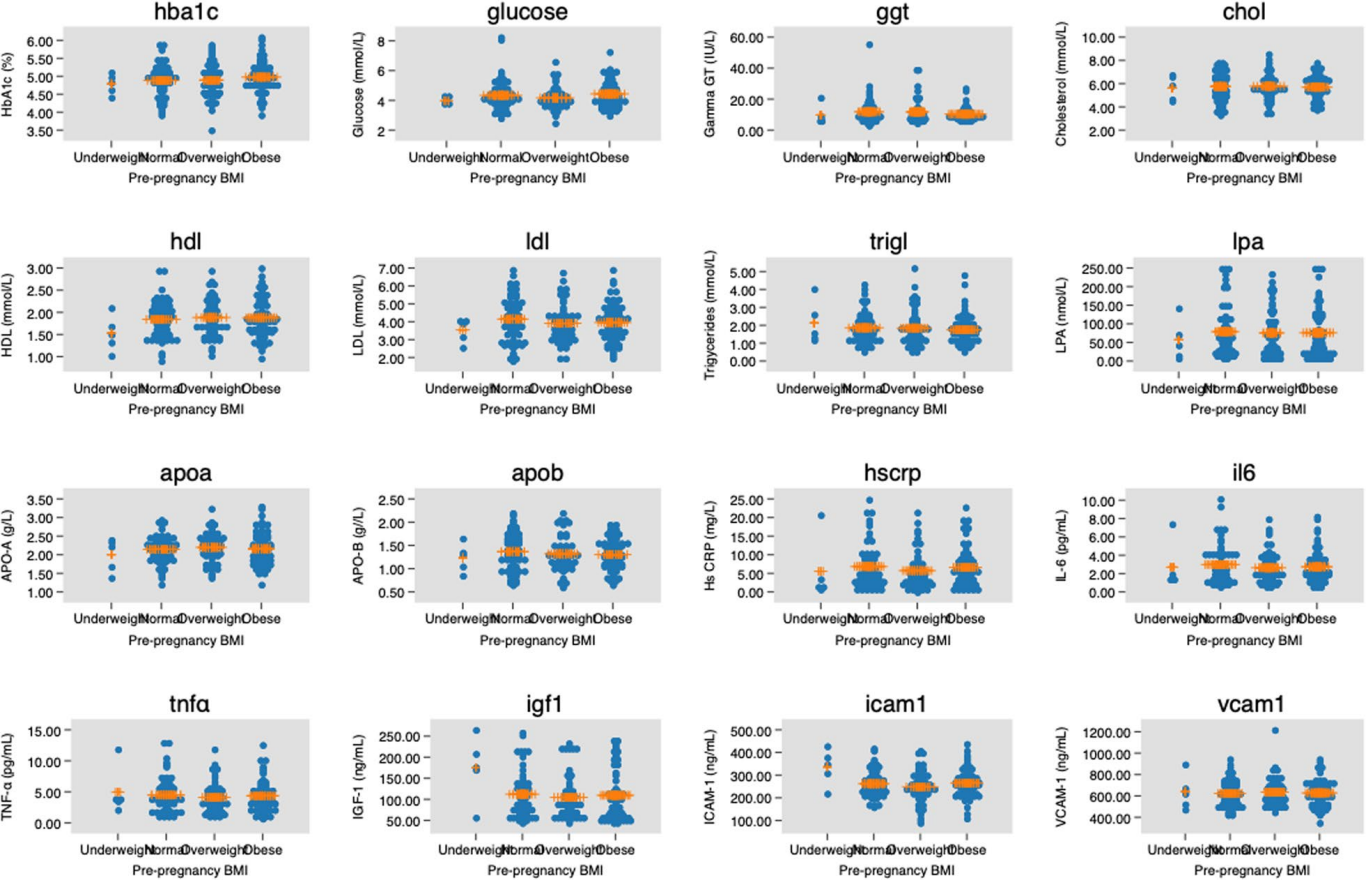
Biomarkers concentrations by pre-pregnancy body mass index (p-bmi) in 178 pregnant women. Horizontal lines indicate mean values and standard deviations

**Fig. 2:**
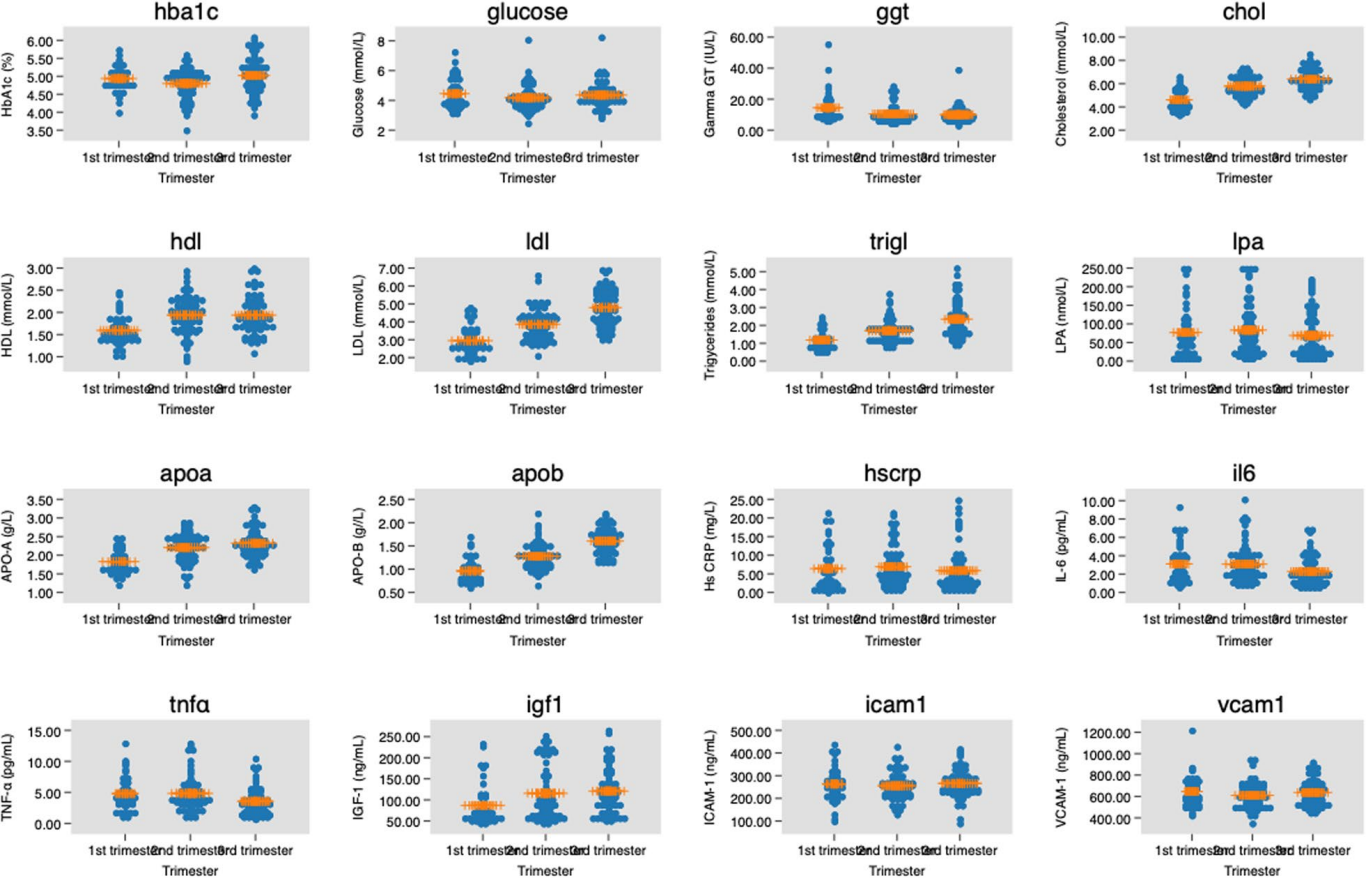
Biomarkers concentrations by trimester of pregnancy in 178 pregnant women. Horizontal lines indicate mean values and standard deviations

Participants’ blood pressure and biomarker measurements across pBMI are presented in Table 2, Fig 1 and Fig 2. None of the blood pressure measurement or biomarkers serum concentrations were statistically different across the pBMI groups except for sICAM-1 levels which were the highest in underweight women with the significant statistically difference observed between them and overweight women in the Bonferroni *post hoc* correction.

**Table 2:** Blood pressure and cardiometabolic biomarkers measurement results (mean ± SD) by pre-pregnancy body mass index classification in 178 pregnant women

Biomarkers	Underweight n = 5	Normal weight n = 59	Overweight n = 52	Obese n = 61	Total n = 178	P-value^a^	Significant differences between^a^	*p*-value^a^
Systolic BP (mmHg)	110 ± 7.9	110 ± 6.9	112 ± 6.8	113 ± 7.8	112 ± 7.3	0.12	NA	NA
Diastolic BP (mmHg)	71 ± 3.6	70 ± 5.04	71 ± 4.83	72 ± 5.2	71 ± 5.0	0.27	NA	NA
HbA_1C_ (%)	4.79 ± 0.28	4.88 ± 0.45	4.89 ± 0.48	4.98 ± 0.43	4.92 ± 0.45	0.55	NA	NA
RBG (mmol/L)	3.97 ± 0.25	4.34 ± 1.01	4.17 ± 0.75	4.43 ± 0.89	4.31 ± 0.88	0.34	NA	NA
GGT (IU/L)	9.78 ± 6.59	11.86 ± 8.07	11.79 ± 7.51	10.32 ± 4.02	11.25 ± 6.69	0.52	NA	NA
Total-C (mmol/L)	5.63 ± 1.09	5.76 ± 1.23	5.79 ± 1.09	5.70 ± 0.99	5.74 ± 0.99	0.97	NA	NA
HDL (mmol/L)	1.53 ± 0.40	1.84 ± 0.42	1.88 ± 0.44	1.88 ± 0.44	1.86 ± 0.43	0.34	NA	NA
LDL (mmol/L)	3.54 ± 0.67	4.15 ± 1.29	3.91 ± 1.08	3.95 ± 1.09	3.99 ± 1.15	0.54	NA	NA
TG (mmol/L)	2.13 ± 1.22	1.86 ± 0.86	1.84 ± 1.00	1.75 ± 0.81	1.82 ± 0.89	0.76	NA	NA
LP-a (nmol/L)	57.20 ± 55.63	78.94 ± 69.26	75.66 ± 63.10	76.12 ± 67.65	76.39 ± 66.17	0.91	NA	NA
APO-A (g/L)	2.00 ± 0.44	2.15 ± 0.37	2.20 ± 0.39	2.16 ± 0.44	2.16 ± 0.40	0.72	NA	NA
APO-B (g/L)	1.23 ± 0.32	1.37 ± 0.40	1.33 ± 0.38	1.30 ± 0.34	1.33 ± 0.37	0.73	NA	NA
Hs-CRP (mg/l)	5.56 ± 8.62	6.83 ± 6.02	5.72 ± 4.94	6.61 ± 5.43	6.39 ± 5.57	0.72	NA	NA
IL-6 (pg/ml)	2.71 ± 2.58	2.99 ± 2.15	2.64 ± 1.76	2.74 ± 1.77	2.79 ± 1.91	0.80	NA	NA
TNF-α (pg/ml)	4.96 ± 3.95	4.50 ± 2.82	4.11 ± 2.47	4.36 ± 2.47	4.351 ± 2.62	0.83	NA	NA
IGF-1 (ng/ml)	175.21 ± 76.92	112.21 ± 55.96	104.83 ± 53.61	100.59 ± 62.42	110.91 ± 58.82	0.08	NA	NA
sICAM-1 (ng/ml)	336.81 ± 78.27	262.27 ± 57.79	248.30 ± 71.09	265.31 ± 62.55	261.34 ± 64.65	0.025	Underweight vs overweight	0.045
sVCAM-1 (ng/ml)	640.62 ± 168.78	623.79 ± 119.88	635.73 ± 132.55	628.08 ± 126.33	629.25 ± 126.26	0.96	NA	NA

Normal weight (BMI = 18.5–24.9 kg/m2), overweight (BMI = 25–29.9 kg/m2), obese (BMI = ≥ 30 kg/m2)

SD: standard deviation; NA: not applicable. BP: blood pressure. HbA_1c_: hemoglobin A_1c_; RBG: Random blood glucose; GGT: gamma-glutamyl transferase; Total-C: total cholesterol; HDL: high-density lipoprotein; LDL: low-density lipoprotein; TG: triglycerides; Lp-a: lipoprotein- a; Apo-A: apolipoprotein-A; Apo-B: apolipoprotein-B; Hs-CRP: high-sensitivity C-reactive protein; IL-6: interleukin-6; TNF-α: tumor necrosis factor-alpha; IGF-1: Insulin-like growth factor-1; sICAM-1: soluble intercellular cytoadhesive molecule-1; sVCAM-1: soluble vascular cytoadhesive molecule-1

^a^One-way analysis of variance (ANOVA) with Bonferroni post hoc correction

**Fig. 3:**
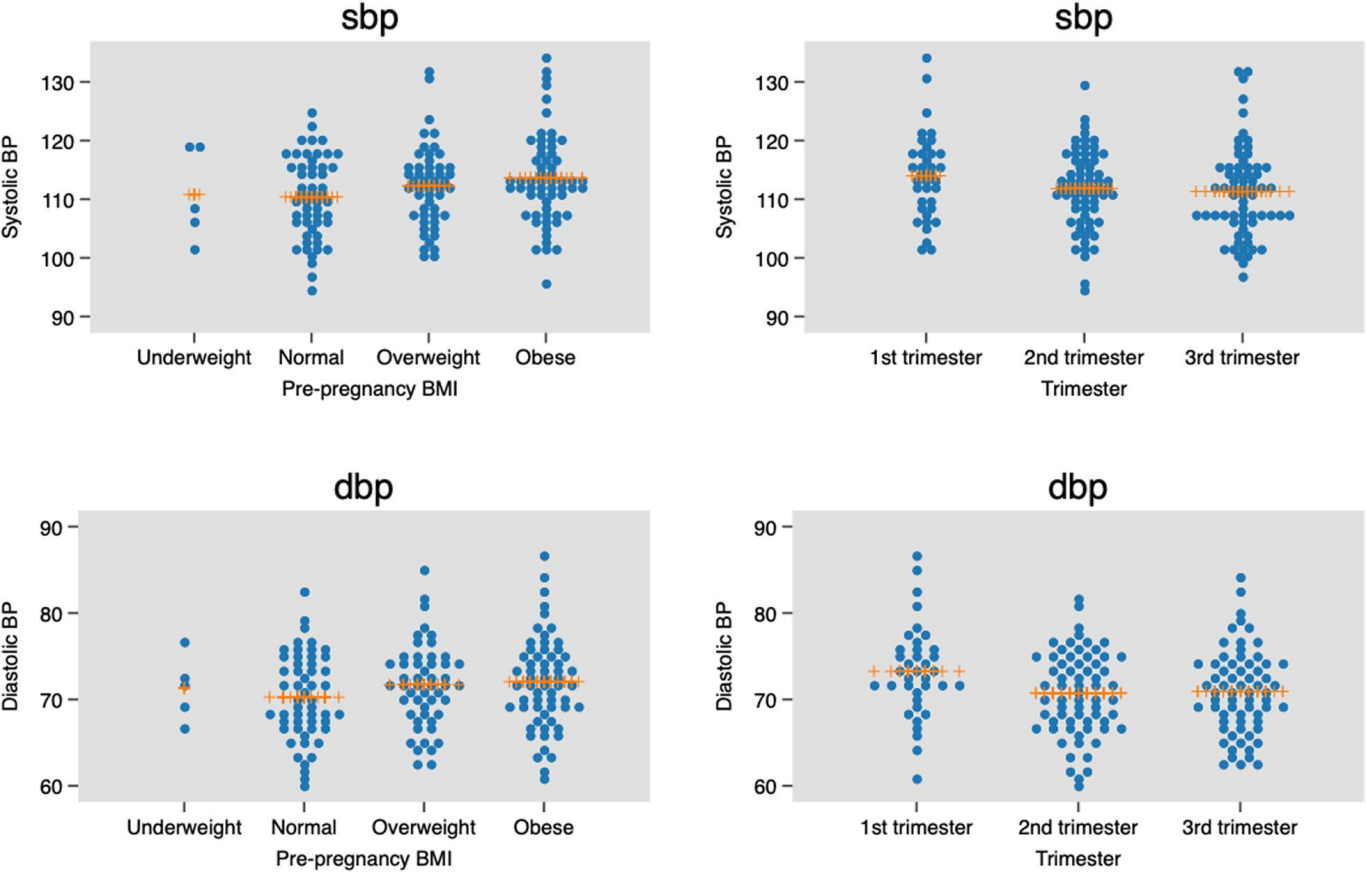
Blood pressure by pBMI and trimester of pregnancy. SBP (systolic blood pressure); DBP (diastolic blood pressure). Horizontal lines indicate mean values and standard deviations

Throughout pregnancy, all variables except systolic blood pressure, random blood glucose (RBG), LP-a, and Hs-CRP were statistically significantly different throughout pregnancy (Table 3, Figs 2 and 3).

**Table 3:** Blood pressure and cardiometabolic biomarkers measurement results (mean ± SD) in 178 pregnant women by trimester of pregnancy

Biomarkers	First trimester n = 42	Second trimester n = 69	Third trimester n = 67	Total n = 178	p-value^a^	Significant differences between^b^	*p*-value^b^
Systolic BP (mmHg)	114 ± 7.3	111 ± 6.8	111 ± 7.7	112 ± 7.3	0.18	NA	NA
Diastolic BP (mmHg)	73 ± 5.34	70 ± 4.9	71 ± 4.8	71 ± 5.0	0.03	1st vs 2nd trimester	0.045
HbA_1C_ (%)	4.94 ± 0.34	4.80 ± 0.41	5.02 ± 0.51	4.92 ± 0.88	0.01	2nd vs 3rd trimester	0.011
RBG (mmol/L)	4.45 ± 1.01	4.18 ± 0.83	4.36 ± 0.83	4.31 ± 0.88	0.25	1st vs 2nd trimester 1st vs 3rd trimester 2nd vs 3rd trimester	< 0.001 < 0.001 < 0.001
GGT (IU/L)	14.46 ± 9.48	10.48 ± 5.52	10.01 ± 4.91	11.25 ± 6.69	0.001	1st vs 2nd trimester	< 0.001
Total-C (mmol/L)	4.61 ± 0.87	5.79 ± 0.81	6.40 ± 0.91	5.74 ± 1.10	< 0.001	1st vs 2nd trimester	< 0.001
HDL (mmol/L)	1.59 ± 0.34	1.94 ± 0.43	1.94 ± 0.42	1.86 ± 0.43	< 0.001	1st vs 2nd trimester 1st vs 3rd trimester 2nd vs 3rd trimester	< 0.001 < 0.001 < 0.001
LDL (mmol/L)	2.94 ± 0.81	3.86 ± 0.85	4.79 ± 1.00	3.99 ± 1.15	< 0.001	1st vs 2nd trimester 1st vs 3rd trimester 2nd vs 3rd trimester	< 0.001 < 0.001 < 0.001
TG (mmol/L)	1.18 ± 0.51	1.70 ± 0.63	2.35 ± 1.00	1.82 ± 0.89	< 0.001	1st vs 3rd trimester	< 0.001
LP-a (nmol/L)	76.95 ± 69.14	83.41 ± 69.41	68.80 ± 60.82	76.39 ± 66.17	0.43	1st vs 2nd trimester 1st vs 3rd trimester 2nd vs 3rd trimester	< 0.001 < 0.001 < 0.001
APO-A (g/L)	1.83 ± 0.31	2.21 ± 0.37	2.32 ± 0.37	2.16 ± 0.40	< 0.001	2nd vs 3rd trimester	0.045
APO-B (g/L)	0.96 ± 0.26	1.28 ± 0.28	1.60 ± 0.28	1.33 ± 0.37	< 0.001	1st vs 3rd trimester 2nd vs 3rd trimester	0.041 0.012
Hs-CRP (mg/l)	6.41 ± 5.88	6.91 ± 5.19	5.85 ± 5.76	6.39 ± 5.57	0.54	1st vs 2nd trimester 1st vs 3rd trimester	0.034 0.009
IL-6 (pg/ml)	3.11 ± 2.08	3.08 ± 2.02	2.29 ± 1.57	2.79 ± 1.91	0.02	2nd vs 3rd trimester	0.011
TNF-α (pg/ml)	4.81 ± 2.72	4.84 ± 2.72	3.56 ± 2.27	4.35 ± 2.62	0.006	1st vs 2nd trimester 1st vs 3rd trimester 2nd vs 3rd trimester	< 0.001 < 0.001 < 0.001
IGF-1 (ng/ml)	86.96 ± 50.13	115.75 ± 65.22	120.93 ± 53.32	110.91 ± 58.82	0.008	1st vs 2nd trimester	< 0.001
sICAM-1 (ng/ml)	263.27 ± 75.07	255.56 ± 60.29	266.09 ± 62.47	261.34 ± 64.65	0.62	NA	NA
sVCAM-1 (ng/ml)	647.07 ± 142.20	610.71 ± 116.89	637.17 ± 124.45	629.25 ± 126.26	0.27	NA	NA

SD: standard deviation; BP: blood pressure; HbA_1c_: hemoglobin A_1c_; RBG: Random blood glucose; GGT: gamma-glutamyl transferase; Total-C: total cholesterol; HDL: high-density lipoprotein; LDL: low-density lipoprotein; TG: triglycerides; Lp-a: lipoprotein- a; Apo-A: apolipoprotein-A; Apo-B: apolipoprotein-B; Hs-CRP: high-sensitivity C-reactive protein; IL-6: interleukin-6; TNF-α: tumor necrosis factor-alpha; IGF-1: Insulin-like growth factor-1; sICAM-1: soluble intercellular cytoadhesive molecule-1; sVCAM-1: soluble vascular cytoadhesive molecule-1; NA: not applicable

^a^One-way analysis of variance (ANOVA) with Bonferroni post hoc correction

**Fig. 4:**
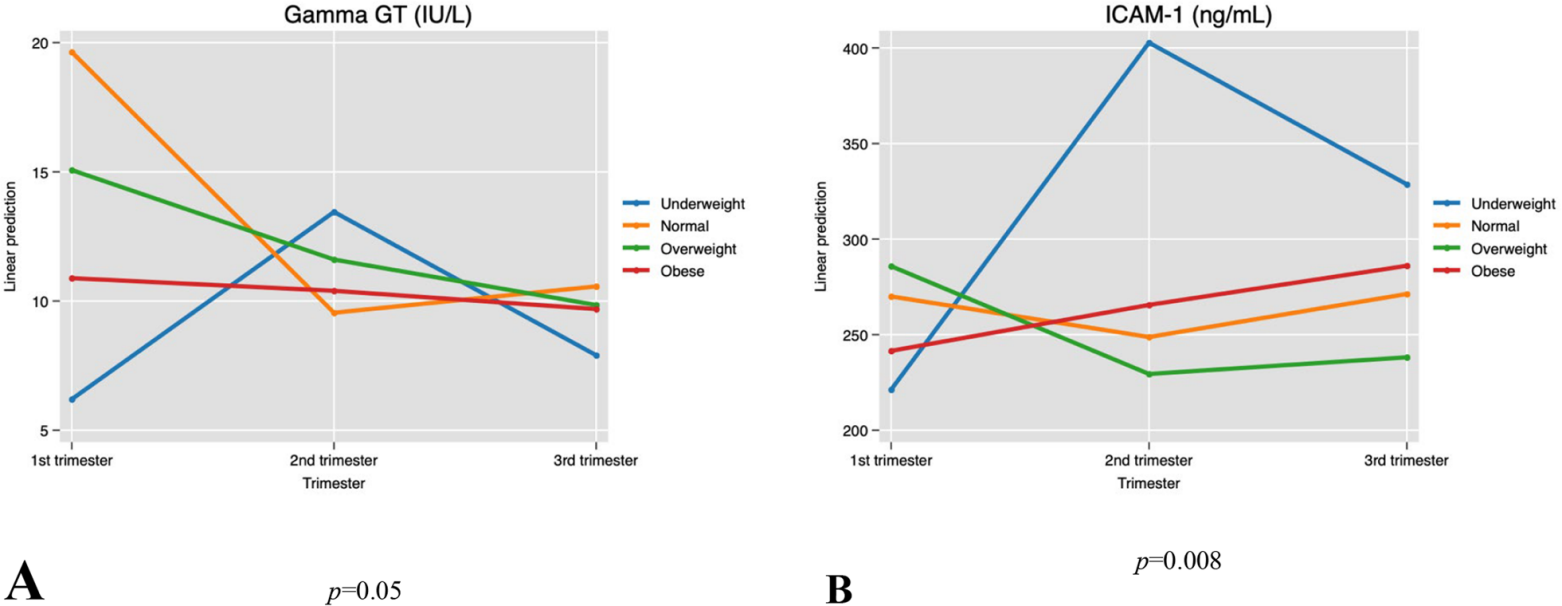
Interaction effect between pBMI and trimester of pregnancy on serum GGT and iCAM-1 concentrations. GGT: gamma-glutamyl transferase, sICAM-1: soluble intercellular cytoadhesive molecule-1. Testing for interaction evaluates the simultaneous effect of trimester of pregnancy and pBMI on GGT (panel **A**) and sICAM-1 (panel **B**), as well and any interaction effect between trimester of pregnancy and pBMI where the effect of one variable depends on the other. Each plot displays the trimesters of pregnancy on the x-axis, the fitted values of GGT and iCAM-1 levels on the y-axis, and separate lines for each category of pBMI. Parallel lines would have indicated the absence of interaction between trimester of pregnancy and pBMI, but the presence of different slopes and nonparallel lines indicate the presence of a statistically significant interaction between trimester of pregnancy and pBMI (*p* = 0.05 and 0.008 respectively), indicating that the effect of trimester on the concentrations of GGT and iCAM-1 is not constant but depends on the value of pBMI

After adjusting, in a multivariate linear regression model, for maternal age, trimester of pregnancy, education level, and parity in a multivariate linear regression model, none of the biomarkers or BP were found to be significantly associated with pBMI. (Table 4). In a multivariate linear regression model adjusting for maternal age, pBMI, education level, parity and smoking, GGT, total-C, DL, LDL, TG, APO-A, APO-B, IL-6, TNF-α and IGF-1 concentrations remained significantly associated with trimester of pregnancy (Table 4). There was a significant interaction between pBMI and trimester of pregnancy only for serum GGT and sICAM−1 concentrations (Table 4, Fig. 4).

**Table 4:** Association between biomarkers concentrations and blood pressure with pre—pregnancy body mass index (pBMI) in 178 pregnant women

	Univariate model^a^		Multivariate model^b^		
Response variable	Coefficient (95% ci)	*p − *value	Coefficient (95% ci)	*p − *value	Interaction between pBMI and trimester of pregnancy (p-value)
Systolic BP (mmHg)	− 1.23 (− 2.68, 0.21)	0.093	0.047 (− 0.14, 0.24)	0.626	NA
Diastolic BP (mmHg)	0.75 (− 0.10, 1.61)	0.085	− 0.003 (− 0.13, 0.13)	0.965	NA
HbA1C (%)	0.050 (− 0.02, 012)	0.184	NA	NA	NA
RBG (mmol/L)	0.006 (− 0.081, 0.21)	0.379	NA	NA	NA
GGT (IU/L)	− 0.56 (− 1.67, 0.55)	0.320	NA	NA	0.05
Total − C (mmol/L)	− 0.02 (− 0.20, 0.16)	0.848	NA	NA	NA
HDL (mmol/L)	0.039 (− 0.03, 0.11)	0.283	NA	NA	NA
LDL (mmol/L)	− 0.05 (− 0.24, 0.14)	0.608	NA	NA	NA
TG (mmol/L)	− 0.07 (− 0.21, 0.78)	0.351	NA	NA	NA
LP − a (nmol/L)	0.175 (− 10.86, 11.21)	0.975	NA	NA	NA
APO − A (g/L)	0.017 (− 0.050, 0.08)	0.621	NA	NA	NA
APO − B (g/L)	− 0.021 (− 0.08, 0.04)	0.507	NA	NA	NA
Hs − CRP (mg/l)	− 0.031 (− 0.96, 0.89)	0.947	NA	NA	NA
IL − 6 (pg/ml)	− 0.098 (− 0.41, 0.22)	0.542	NA	NA	NA
TNF − α (pg/ml)	− 0.09 (− 0.53, 0.33)	0.656	NA	NA	NA
IGF − 1 (ng/ml)	− 5.69 (− 15.47, 4.07)	0.252	NA	NA	NA
sICAM − 1 (ng/ml)	− 4.00 (− 14.77, 6.76)	0.464	NA	NA	0.008
sVCAM − 1 (ng/ml)	0.966 (− 20.09, 22.26)	0.867	NA	NA	NA
Systolic BP (mmHg)	− 1.23 (− 2.68, 0.21)	0.093	0.047 (− 0.14, 0.24)	0.626	NA
Diastolic BP (mmHg)	0.75 (− 0.10, 1.61)	0.085	− 0.003 (− 0.13, 0.13)	0.965	NA

ci: confidence intervals; BP: blood pressure; HbA1c: hemoglobin A_1c_; RBG: Random blood glucose; GGT: gamma-glutamyl transferase; Total-C: total cholesterol; HDL: high-density lipoprotein; LDL: low-density lipoprotein; TG: triglycerides; Lp-a: lipoprotein-a; Apo-A: apolipoprotein-A; Apo-B: apolipoprotein-B; Hs-CRP: high-sensitivity C-reactive protein; IL-6: interleukin-6; TNF-α: tumor necrosis factor-alpha; IGF-1: Insulin-like growth factor-1; sICAM-1: soluble intercellular cytoadhesive molecule-1; sVCAM-1: soluble vascular cytoadhesive molecule-1; NA: not applicable

^a^Linear regression model

^b^linear regression adjusting for maternal age, trimester of pregnancy, education level, and parity

## Discussion

Pregnancy-related significant hormonal and metabolic changes can lead to a state of hyperlipidemia, prothrombosis, pro-inflammatory, and extreme insulin resistance [31]. Furthermore, early pregnancy’s elevated levels of progesterone, cortisol, and estrogen encourage the production of fat and lipogenesis [32]. A person may be more susceptible to developing metabolic syndrome as a result of these alterations Table 5.

**Table 5:** Association between blood pressure and biomarkers concentrations with trimester of pregnancy in 178 pregnant women

Response variable	Univariate model^a^		Multivariate model^b^		Interaction between pBMI and trimester of pregnancy
	Coefficient (95% ci)	*p-*value	Coefficient (95% ci)	*p-*value	*p-*value
Systolic BP (mmHg)	− 1.23 (− 2.68, 0.208)	0.093	− 1.08 (− 2.74, 0.58)	0.21	NA
Diastolic BP (mmHg)	− 1.00 (− 2.00, − 0.006)	0.049	− 1.11 (− 2.27, 0.053)	0.061	NA
HbA_1C_ (%)	− 0.05 (− 0.27, 0.14)	0.184	NA	NA	NA
RBG (mmol/L)	− 0.024 (− 0.19, 0.14)	0.779	NA	NA	NA
GGT (IU/L)	− 2.06 (− 3.31, − 0.81)	0.001	− 1.67 (− 2.96, − 0.38)	0.011	0.05
Total-C (mmol/L)	0.87 (0.70, 1.04)	< 0.001	0.97 (0.78, 1.16)	< 0.001	NA
HDL (mmol/L)	0.15 (− 0.07, 0.23)	< 0.001	0.18 (0.09, 0.27)	< 0.001	NA
LDL (mmol/L)	0.92 (0.75, 1.09)	< 0.001	1.03 (0.84, 1.22)	< 0.001	NA
TG (mmol/L)	0.59 (0.42, 0.76)	< 0.001	0.59 (0.42, 0.76)	< 0.001	NA
LP-a (nmol/L)	− 5.04 (− 17.7, 7.68)	0.435	NA	NA	NA
APO-A (g/L)	0.23 (0.16, 0.30)	< 0.001	0.23 (0.16, 0.31)	< 0.001	NA
APO-B (g/L)	0.32 (0.26, 0.37)	< 0.001	0.35 (0.29, 0.41)	< 0.001	NA
Hs-CRP (mg/l)	− 0.03 (− 0.96, 0.89)	0.947	NA	NA	NA
IL-6 (pg/ml)	− 0.44 (− 0.80, − 0.08)	0.016	− 0.49 (− 0.88, − 0.092)	0.016	NA
TNF-α (pg/ml)	− 0.68 (− 1.18, − 0.19)	0.007	− 0.77 (− 1.31, − 0.22)	0.006	NA
IGF-1 (ng/ml)	15.90 (4.82, 26.99)	0.005	15.39 (2.91, 27.87)	0.016	NA
sICAM-1 (ng/ml)	2.24 (− 10.20, 14.70)	0.722	NA	NA	0.008
sVCAM-1 (ng/ml)	− 2.06 (− 26.38, 22.26)	0.867	NA	NA	NA

ci: confidence intervals; BP: blood pressure; HbA1c: hemoglobin A_1c_; RBG: Random blood glucose; GGT: gamma-glutamyl transferase; Total-C: total cholesterol; HDL: high-density lipoprotein; LDL: low-density lipoprotein; TG: triglycerides; Lp-a: lipoprotein- a; Apo-A: apolipoprotein-A; Apo-B: apolipoprotein-B; Hs-CRP: high-sensitivity C-reactive protein; IL-6: interleukin-6; TNF-α: tumor necrosis factor-alpha; IGF-1: Insulin-like growth factor-1; sICAM-1: soluble intercellular cytoadhesive molecule-1; sVCAM-1: soluble vascular cytoadhesive molecule-1; NA: not applicable

^a^Linear regression model

^b^linear regression adjusting for maternal age, pre-pregnancy body mass index, education level

The goal of the current study was to determine how changes in maternal biomarkers during pregnancy increased the risk of metabolic disorders in obese women. The current study revealed statistically significant changes in hemodynamic and metabolic markers throughout pregnancy.

It has been demonstrated that pBMI is a predictive value for abnormal concentration of cardiometabolic markers during pregnancy, which have been linked to adverse effects on both maternal cardiovascular system and perinatal outcomes [1–7, 12].

Although not achieving statical significance, this study reveals some associations between pBMI, pregnancy trimesters, cardiometabolic markers, and BP levels. We did not observe a statistically significant association between overweight women and elevated levels of systemic inflammatory markers during pregnancy compared to those with lower or higher pBMI, in contrast to previous studies that have shown different results [1, 3, 6, 7, 12, 14–17]. Women who were either pre-gestational overweight or obese did not exhibited higher levels of IL-6 and TNF-α compared to other pBMI groups, although not achieving statistical significance, contrasting with results of a previous meta-analysis [33]. The biological reasons for these differences remain unclear.

Regardless of pBMI, we found that advanced pregnancy, particularly during third trimester, although not achieving statical significance was associated with increased concentrations of lipid levels, HbA1c, Apo A and B, but not LP-a, confirming previous studies [10–14, 16, 34]. HbA_1c_ levels were increased in the third trimester and decreased in the second trimester compared to the first trimester, confirming previous reports. This variation can be attributed to physiological changes in blood cell concentration during pregnancy [10]. Although, it has been shown that pre-gestational obesity is associated with elevated blood glucose during pregnancy, our study randomly measured these biomarkers only once during each trimester, precluding our understanding of their trajectory in individual pregnant women throughout entire pregnancy. Furthermore, this study revealed an association, although mot statistically significant, between increasing p-BMI and higher systolic and diastolic blood pressure in pregnant women, confirming previous studies [3, 12, 13, 19, 24, 34].

The novel finding of a statistically significant interaction effect between pBMI and trimester of pregnancy, as main effects, on the levels of GGT and ICAM-1 urges caution in the interpretation of these results. It would thus be incorrect to interpret separately the concentration of each of these biomarkers in each trimester or for each pBMI group (main effects) but to interpret them instead and exclusively by their interaction effect, as their respective concentration varies simultaneously with the pBMI and the trimester when the blood was collected. The relationship between pBMI and GGT or ICAM-1 levels changes depending on the trimester of pregnancy and vice versa [35, 36].

Strengths of this study include focusing only on maternal pBMI in association with the various biomarkers, removing possible confounding effect by other comorbidities, such cardiovascular or renal risk factors or diabetes. The analysis of interaction effect between pBMI and trimester of pregnancy is useful as for the involved biomarkers, the significance of their serum levels cannot be interpreted according to pBMI or trimester of pregnancy, but only by the predicted values obtained with their interaction. Such analysis has been lacking in many previous reports, therefore making their interpretation and the resulting recommendations suboptimal.

We acknowledge that a potential selection bias may exist in this study, as women attending these two antenatal clinics may not be fully representative of general population. It is also possible that women who are obese or have other comorbidities may be more inclined to seek medical care during pregnancy, which could introduce a selection bias. Furthermore, measurement bias cannot be completely ruled out, as assessment of cardiometabolic biomarkers can be influenced by various factors, including timing of blood sampling, fasting status and variations throughout pregnancy. An important limitation is that no longitudinal trajectory of the biomarkers was made serially in each pregnant woman, but blood sampling was performed only once in each woman randomly in each trimester of pregnancy, similar to three separate descriptive cohort studies, one in each trimester of pregnancy. Furthermore, this study did not look at morbidity and mortality rates in both mothers and offspring and their association with pBMI or maternal cardiometabolic biomarkers. We also acknowledge that there are several confounding factors that were not included although they could potentially influence the association between pBMI and maternal cardiometabolic biomarkers. These include socioeconomic status, physical activity and diet, which have been shown to also have an impact, but unfortunately were not evaluated in present study. Additionally, it is necessary to consider possibility of reverse causation, as relationship between pBMI and maternal cardiometabolic biomarkers may be bidirectional: it is equally plausible that elevated levels of cardiometabolic biomarkers could contribute to obesity, or conversely, obesity could influence cardiometabolic biomarkers levels. We acknowledge that generalizability of these findings may be limited, as association between pBMI and maternal cardiometabolic biomarkers could vary among different racial/ethnic groups and geographical regions. The lack of statistical difference may be attributed to the limited sample size, the inclusion of only Emirati women, the exclusion of pregnant women known to be hypertensive or diabetic, or with a multiple pregnancy.

While this study provides valuable evidence of an association and interaction effect, the design does not allow for establishing causality, and therefore, evidence-based recommendations cannot be made solely based on these results. Future studies, with larger sample size and serial measurements of biomarkers in each individual participant throughout pregnancy, should strive to address our limitations in order to gain a better understanding of relationship between pBMI and maternal cardiometabolic health during pregnancy.

## Conclusions

While this study does not establish causality, it highlights the hyperlipidemic state of pregnancy and the diabetogenic tendency in obese, non-diabetic pregnant women without other pre-existing risk factors for metabolic syndrome. These findings suggest that pregnancy in obese women may contribute to an elevated risk of developing metabolic syndrome. However, further research through larger longitudinal cohort studies with serial biomarker measurements in obese women throughout pregnancy is essential. Such studies could uncover the underlying mechanisms driving these associations and inform the development of targeted intervention strategies to identify significant cardiometabolic risk factors and prevent or mitigate serious health complications for both mother and fetus.

## Data Availability

No datasets were generated or analysed during the current study.

## Abbreviations

pBMI: Pre-pregnancy body mass index
BP: Blood pressure
HbA1c: Hemoglobin A1c
RBG: Random blood glucose
GGT: Gamma-glutamyl transferase
total-C: Total cholesterol
HDL: High-density lipoprotein
LDL: Low-density lipoprotein
TG: Triglycerides
Lp-a: Lipoprotein- a
Apo-A: Apolipoprotein-A
Apo-B: Apolipoprotein-B
Hs-CRP: High-sensitivity C-reactive protein
IL-6: Interleukin-6
TNF-α: Tumor necrosis factor-alpha
IGF-1: Insulin-like growth factor-1
sICAM-1: Soluble intercellular cytoadhesive molecule-1
sVCAM-1: Soluble vascular cytoadhesive molecule-1
